# Predictive value of serum apolipoprotein panel (ApoA1 / ApoA2 / ApoA4) as a biomarker for individual radiosensitivity

**DOI:** 10.1186/s12944-026-02868-8

**Published:** 2026-01-26

**Authors:** Na Huang, Heming Wang, Xiao Li, Yuhong Xiang, Ziteng Liu, Yaqiong Li, Hongmei Zhou, Qi Wang, Hongwei Zhou, Zhenhua Qi, Zhidong Wang

**Affiliations:** 1 Beijing Institute of Radiation Medicine, Beijing, 100850 China; 2 Department of General Medicine, The Fourth Medical Center of Chinese PLA General Hospital, Beijing, 100048 China; 3 Graduate Collaborative Training Base of Academy of Military Sciences, Hengyang Medical School, University of South China, Hengyang, Hunan 421001 China

**Keywords:** Cholesterol metabolism, Apolipoprotein panel (ApoA1/ApoA2/ApoA4), Individual radiosensitivity, Predictive model, Biomarker, High-throughput proteomics, XGBoost machine learning

## Abstract

**Background:**

Significant interindividual variability in radiosensitivity poses a major challenge to conventional radiation protection and radiotherapy. Current prediction strategies relying on DNA damage or genomic analysis have inherent limitations, underscoring the need for minimally invasive serum biomarkers. While serum apolipoproteins are crucial regulators of lipid transport, metabolism, and cellular stress response, their role as biomarkers for radiosensitivity remains largely unexplored.

**Methods:**

A 7.3 Gy ⁶⁰Co γ-ray whole-body irradiation mouse model (with training and independent validation cohorts) was established to assess individual radiosensitivity. Pre-irradiation peripheral serum samples underwent high-throughput proteomics analysis to identify differential proteins (DEPs) linked to 30-day post-irradiation survival. KEGG and GO enrichment analyses were conducted to characterize DEP-associated pathways. An XGBoost machine learning model was built using candidate biomarkers, with SHAP analysis to define their predictive contributions; Cox proportional hazards and Pearson correlation analyses were applied to evaluate survival associations.

**Results:**

DIA-based proteomics identified 580 DEPs in the training cohort and 449 in the validation cohort. KEGG and GO enrichment analyses confirmed that these DEPs were predominantly enriched in the cholesterol metabolism and reverse cholesterol transport pathways. The predictive model based on an apolipoprotein panel (ApoA1/ApoA2/ApoA4), established using the XGBoost algorithm, exhibited exceptional performance in the training cohort (AUC = 1) and maintained robust generalizability in an independent validation cohort (AUC = 0.833). Compared with non-survivors, survivors exhibited significantly elevated serum levels of ApoA1 and ApoA2 but markedly reduced levels of ApoA4. Cox proportional hazards regression analysis established ApoA1 and ApoA2 as independent protective factors, whereas high ApoA4 expression was an adverse prognostic indicator. Notably, ApoA4 levels also demonstrated a strong negative correlation with post-irradiation survival time.

**Conclusion:**

The serum apolipoprotein profile (ApoA1/ApoA2/ApoA4) serves not only as a promising minimally invasive biomarker for predicting individual radiosensitivity in mice but also reveals a critical link between the cholesterol metabolic pathway and radiation response. This finding lays a theoretical foundation for translating predictive, cholesterol metabolism-related biomarkers to support radiation response assessments. Given the limitations of animal models, subsequent studies are required to validate the clinical applicability of this panel in human cohorts, with the aim of offering an effective tool for personalized radiation protection and precise radiotherapy.

**Supplementary Information:**

The online version contains supplementary material available at 10.1186/s12944-026-02868-8.

## Background

Individual radiosensitivity, a critical determinant of health outcomes following radiation exposure, exhibits substantial interindividual variation with severe consequences for diverse populations [1]. These groups include personnel involved in nuclear emergency response, occupational exposure cohorts (e.g., nuclear medicine personnel), and tumor patients undergoing radiotherapy. This variability is well-documented in epidemiological studies: long-term follow-up of Chernobyl cleanup workers, for instance, demonstrated that emergency personnel carrying radiosensitivity-associated genetic markers faced a significantly higher risk of radiation-related diseases (e.g., hematological malignancies and chronic ischemic heart disease) compared to their counterparts without such markers [2]. Further evidence comes from a comprehensive meta-analysis of 6,682 cancer patients undergoing radiotherapy, which confirmed that the XRCC1 rs25487 (Arg399Gln) polymorphism, a key defect in the base excision repair pathway, significantly increases the risk of acute radiation-induced toxicity (specifically mucositis and gastrointestinal effects) [3]. Consequently, the prediction and assessment of individual radiosensitivity are pivotal to advancing radiation safety and protection systems and optimizing nuclear medicine outcomes. These goals can be achieved through three key strategies: prescreening for hypersensitive individuals, dynamic biological dose monitoring of occupational personnel, and personalized radiotherapy regimens tailored to interindividual tumor radiosensitivity [4, 5].

Individual variation in radiosensitivity is a complex phenotype shaped by genetics, age, sex, and other factors. Mechanistically, this variation stems from the aberrant expression or function of key genes that regulate core biological processes, including DNA damage repair, cell cycle checkpoint control, and metabolism (e.g., lipid metabolism) [6–8]. This is exemplified in genetic disorders like ataxia-telangiectasia (ATM mutation), Ligase IV syndrome (LIG4 mutation), and Nijmegen breakage syndrome (NBS1 mutation), where patients exhibit extreme radiosensitivity [9]. Furthermore, common genetic variants, such as the methionine variant at position 241 of the XRCC3 gene, while not causative of disease, can enhance individual radiosensitivity by impairing DNA repair protein function [10, 11]. Additionally, deficiency in apolipoproteins such as ApoE, by impairing cholesterol transport and leading to the accumulation of DNA oxidative damage in organs, significantly enhances individual radiosensitivity [12, 13]. Therefore, based on these molecular mechanisms, developing biomarkers to predict individual radiosensitivity has become a pivotal research endeavor for advancing both radiation protection and precision radiotherapy.

Biomarkers of radiosensitivity can indicate individual differences and provide insights into radiation-induced biological processes. Traditional methods based on DNA damage (e.g., chromosomal aberrations, micronucleus formation) have been used in monitoring nuclear workers and astronauts. However, these approaches are significantly limited by time-consuming assays that require skilled personnel [14, 15]. Similarly, while genomic tests (e.g., for ATM, TP53) can reveal genetic susceptibility, they face challenges including high cost, long turnaround time, and reliance on static genetic information [5]. Consequently, there is an urgent need for novel biomarkers that are rapid, simple, and efficient to overcome these constraints. In recent years, numerous studies have focused on discovering intrinsic radiation biomarkers in serum or plasma, such as proteins and non-coding RNAs. Serum protein detection holds great promise as a minimally invasive biomarker, given its advantages in operationally convenience and dynamic monitoring [16]. Among candidate proteins, apolipoproteins —core mediators of lipid metabolism—have emerged as promising targets due to their well-established clinical relevance. High-density lipoprotein (HDL), with apolipoproteins (e.g., ApoA1, ApoA2) as key functional components, mediates reverse cholesterol transport; both this metabolic process and associated apolipoproteins have demonstrated utility in the auxiliary diagnosis of autoimmune diseases and malignancies. Notably, accumulating evidence links serum apolipoprotein levels to cancer radiotherapy outcomes. A representative study on nasopharyngeal carcinoma revealed that a significantly elevated ApoB/ ApoA1 ratio following radiotherapy was positively correlated with larger radiation-induced brain necrosis volumes and an elevated risk of diminished quality of life [17]. However, a critical gap persists: existing studies primarily validate the prognostic value of post-radiotherapy apolipoprotein changes, rather than their predictive role in pre-treatment intrinsic radiosensitivity—limiting clinical translation of these findings.

To address these shortcomings, this study employed high-throughput proteomics to systematically screen the pre-irradiation serum protein expression profiles in C57BL/6J mice. For the first time, we constructed an XGBoost-based radiosensitivity prediction model using ApoA1/ApoA2/ApoA4, which exhibited not only excellent predictive performance but also robust generalizability. Cox regression analysis confirmed ApoA1 and ApoA2 as independent protective factors, whereas high ApoA4 expression was identified as an adverse prognostic indicator. Our findings highlight the potential of this apolipoprotein panel as a predictive biomarker for individual radiosensitivity. This evidence establishes a preliminary foundation for advancing occupational radiation protection and cancer precision radiotherapy.

## Methods

### Mouse husbandry

This study utilized specific pathogen-free male C57BL/6J mice (8–10 weeks old, 22–25 g) obtained from Sibeifu (Beijing) Biotechnology Co., Ltd. All mice were housed in the barrier facility of the Experimental Animal Center at the Academy of Military Medical Sciences. All animal procedures were approved by the Institutional Animal Care and Use Committee of Beijing Military Medical Research Institute (Approval No.: IACUC-DWZX-2020-551). The mice were acclimated for 3–4 days prior to any experimental procedures. They were maintained under standard conditions at a temperature of 18–23 °C, humidity of 40%–60%, and a 12/12-hour light/dark cycle, with uniform caging and ad libitum access to food and water.

### Irradiation and grouping

Following the acclimation period, a baseline blood sample (100 µL) was collected from the tail vein 7 days prior to irradiation. The blood was centrifuged to isolate 30 µL of serum, after which the mice were allowed to recover for 7 days. Subsequently, a pilot experiment was conducted to determine the 30-day median lethal dose (LD₅₀/₃₀) of C57BL/6J mice as the basis for selecting the irradiation dose in formal experiments. Specifically, mice were subjected to whole-body ⁶⁰Co γ-ray irradiation at doses of 7 Gy, 7.5 Gy, and 8 Gy at the Cobalt Source Facility of the Institute of Radiation Medicine (see Supplementary Figure S2). The 30-day survival rates of the three irradiation groups were 70%, 20%, and 0%, respectively, indicating that the LD₅₀/₃₀ of C57BL/6J mice falls within the range of 7–7.5 Gy, and thus 7.3 Gy was identified as an optimal dose close to the median lethal level. In the formal irradiation model, all mice received whole-body irradiation with 7.3 Gy of ⁶⁰Co γ-rays at the Cobalt Source Facility of the Institute of Radiation Medicine to establish the radiation model. An irradiation model was established using a group of mice (*n* = 60) that were irradiated at a dose rate of 61.54 mGy/min as the training cohort. Subsequently, in an independent experiment, another group of mice (*n* = 15) that were irradiated at a dose rate of 60.75 mGy/min was used as the validation cohort to verify the model. During irradiation, mice were humanely restrained in transparent, ventilated organic glass boxes. Body weight was recorded one day before irradiation and on days 1, 4, 7, 11, 15, 20, 25, and 30 post-irradiation. Survival was monitored daily throughout the 30-day observation period. At the end of the study, all surviving animals were euthanized. Specifically, C57BL/6 mice were euthanized via intraperitoneal injection of saline-diluted Euthasol at a dose of 150 mg/kg body weight (Shanghai Yuansi Standard Science and Technology Co., Ltd., China), in accordance with the *AVMA Guidelines on Euthanasia* and the methodology described in previous reports [18]. Based on the 30-day survival outcomes, each cohort was further stratified into a radiation-sensitive group (non-survivors) and a radiation-resistant group (survivors). To rule out the potential confounding effect of initial body weight on survival, Pearson correlation analysis was performed, confirming no significant association between body weight on the day of irradiation and survival time.

### Serum sample collection‌

After the 3–4 day acclimation period, baseline blood (100 µL) was collected from the tail vein. After collection, the blood sample was first centrifuged at 4 °C, 5000 rpm for 5 min. The supernatant was then collected and subjected to a second centrifugation at 4 °C, 12,000 rpm for 5 min to obtain 30 µL of serum. The processed serum samples were aliquoted and stored at -80 °C for subsequent proteomic analysis.

### Serum proteomic analysis

Serum proteomic analysis was performed by Shanghai Bioprofile Biotechnology Co., Ltd. using a Thermo Scientific Orbitrap Astral mass spectrometer coupled with a Vanquish Neo UHPLC system for Data-Independent Acquisition (DIA). Samples were sequentially processed for denaturation, reduction, and alkylation, followed by digestion with trypsin. The resulting peptides were desalted and separated on a µPAC™Neo column using an acetonitrile gradient. The separated peptides were ionized and analyzed in DIA mode. Raw data were processed using DIA-NN 1.8 software, searching against the UniProtKB Mus musculus database. The false discovery rate (FDR) was set to 1% at both the peptide-spectrum-match and protein levels. Differential expression analysis between the radiation-sensitive group (mice that died within 30 days) and the radiation-resistant group (mice that survived 30 days) was performed using the Mann-Whitney U test. Proteins with *P* < 0.05 and |log_2_FC| ≥ 0.585 were considered significantly differential for subsequent functional enrichment analysis.

### Functional enrichment analysis‌

To investigate the biological processes and signaling pathways associated with radiosensitivity, Kyoto Encyclopedia of Genes and Genomes (KEGG) and Gene Ontology Biological Process (GO-BP) enrichment analyses were performed on the significantly differential proteins (*P* < 0.05, |log_2_FC| ≥ 0.585). KEGG pathway enrichment analysis was conducted using the clusterProfiler package, with Fisher’s exact test for statistical significance and FDR correction (*P* < 0.05). GO-BP analysis was performed similarly. The top 20 most significantly enriched pathways or biological processes were visualized using ggplot2.

### XGBoost-Based screening for radiosensitivity predictors

To identify key proteins predictive of radiosensitivity, an XGBoost machine learning model was applied to the serum proteins enriched in the most significantly altered pathway (Cholesterol metabolism). The proteomic expression data from the training cohort served as the training set, while data from the validation cohort served as the test set. All data were normalized to mitigate the influence of feature scales. The model was built in R (v4.4.2) using the xgboost package (v1.7.8) with the following core parameters: learning rate (eta) = 0.1, max depth = 6, subsample = 1, min child weight = 1, and nrounds = 100. To mitigate potential overfitting, 5-fold cross-validation was implemented during training—splitting the dataset into five subsets, using four for training and one for independent validation per iteration, evaluating hyperparameters on validation subsets, and retaining only models with consistently robust performance across all subsets. Model performance was evaluated by the Area Under the Receiver Operating Characteristic curve (AUC), with 2000 bootstrap resamples used to calculate the 95% confidence interval. Proteins ranking in the top 10% by XGBoost Gain score and passing a permutation test (*P* < 0.01) were defined as key predictors.

### SHAP analysis

SHapley Additive exPlanations (SHAP) analysis can not only evaluate the overall impact of key predictive proteins on model output from a global perspective but also reveal the direction (promotion or inhibition) and intensity of the predictive value of specific proteins across different samples from a local perspective. To interpret the contribution of each key predictive protein to the model’s output, we performed SHAP analysis targeting candidate serum proteins enriched in the cholesterol metabolism pathway and commonly differentially expressed between the radiosensitive and radioresistant groups in the training cohort. This analysis quantifies the marginal contribution of each protein to the prediction for each individual sample, resulting in a sample-by-protein SHAP value matrix. In this matrix, a positive SHAP value indicates the protein’s positive contribution to the prediction, whereas a negative value suggests an inhibitory effect. The mean absolute SHAP value was used to quantify the global importance of each protein. The relationship between protein expression levels and SHAP-derived directional impact was visualized using a SHAP beeswarm plot.

### Cox proportional-hazards model analysis‌

To further assess the predictive value of key protein expression levels for survival outcomes, we performed Kaplan-Meier survival analysis, stratifying mice from both cohorts into high- and low-expression groups based on the median baseline expression level. Subsequently, a Cox proportional-hazards model was employed to estimate the hazard ratio (HR). Based on R (v4.4.2), the survfit() function was used to construct the model, outputting the HR, its 95% confidence interval, and the coefficient P-value. An HR > 1 indicates increased risk in the high-expression group, while an HR < 1 suggests a protective effect. The log-rank test was performed using the survdiff() function to compare survival curves between groups.

### Statistical analysis‌

Pearson correlation coefficients were calculated to assess linear relationships between pre-irradiation body weight and survival time, as well as between protein expression and survival time, with *P* < 0.05 considered statistically significant. Data are presented as mean ± standard deviation. Intergroup comparisons of protein expression levels were performed using the Mann-Whitney U test. Statistical significance is denoted as *P < 0.05, **‌P < 0.01, ***‌P < 0.001, and ****‌‌P < 0.0001. All statistical analyses and visualizations were performed using GraphPad Prism statistical software V.9.5 (GraphPad Software) and R statistical software V.4.4.2 (R Project for Statistical Computing).

## Results

### Establishment of an irradiated mouse model for evaluating individual radiosensitivity

**Fig. 1:**
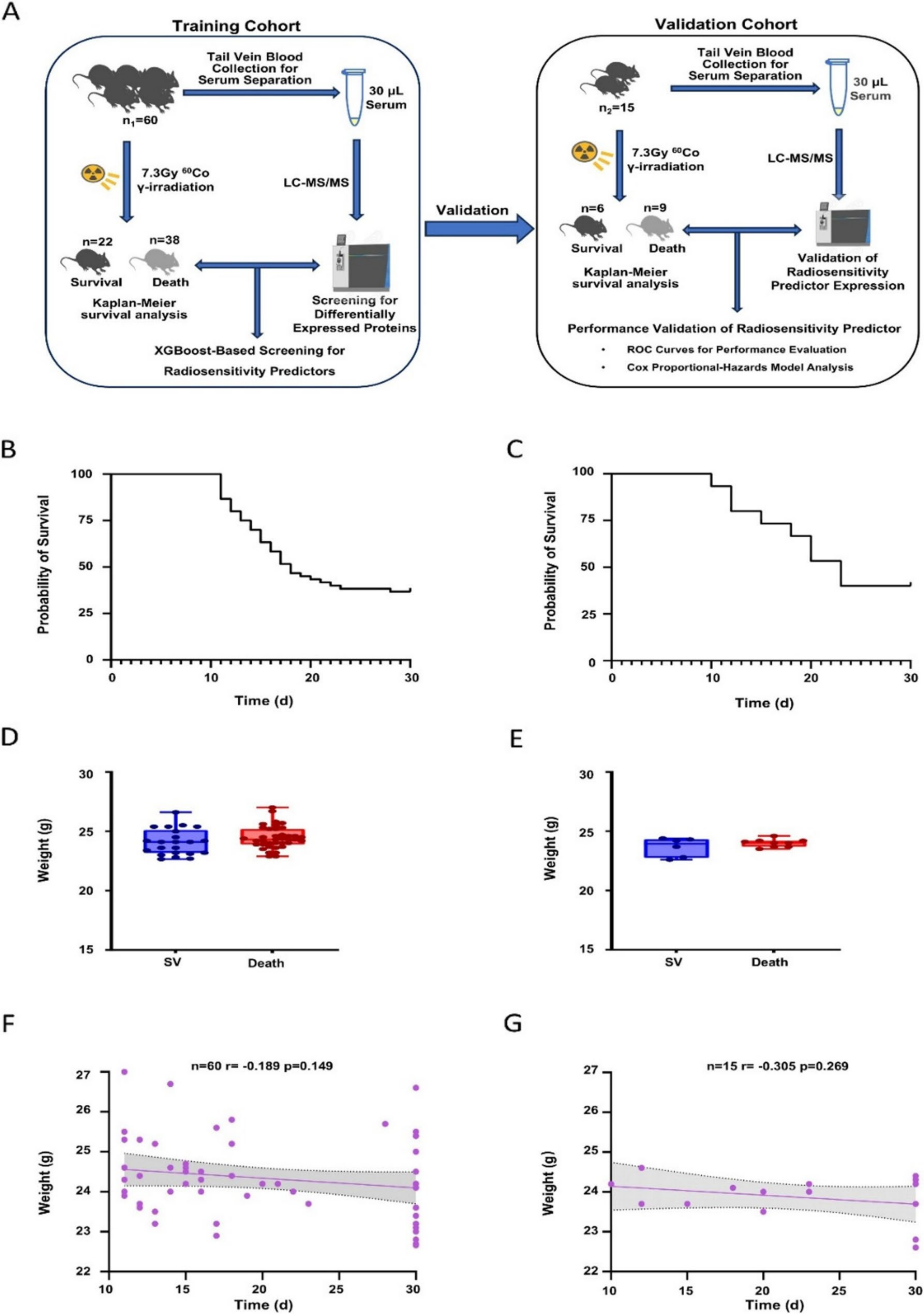
Establishment of a mouse model exposed to the 7.3 Gy median lethal dose.‌ **A**. Workflow of the study. **B**-**C**. Kaplan-Meier 30-day survival curves for mice in the training cohort (*n* = 60) and validation cohort (*n* = 24). **D**-**E**. Box plots of the initial body weight before irradiation for mice in the training and validation cohorts (SV: survival group; Death: death group; unit: g). **F**-**G**. Pearson correlation analysis between initial body weight and post-irradiation survival time in the training and validation cohorts

To assess differences in individual radiosensitivity, this study established a median lethal dose model by subjecting C57BL/6J mice to 7.3 Gy ⁶⁰Co γ-ray whole-body irradiation. Two independent experiments were conducted to form a training cohort (*n* = 60) and a validation cohort (*n* = 15), respectively. The 30-day survival rate was recorded post-irradiation, and pre-irradiation peripheral serum samples were subjected to high-throughput proteomic analysis to examine the correlation between baseline peripheral serum protein expression and mouse survival. Serum samples from the training cohort were used for differential protein screening and machine learning model construction, while those from the validation cohort were used for independent verification of key protein biomarkers, ensuring the reliability of the findings (Fig. 1A).

The results showed a 30-day survival rate of 36.7% in the training cohort (22 survived, 38 died) and 40.0% in the validation cohort (6 survived, 9 died) (Fig. 1B and C). Additionally, no significant statistical differences in initial body weight were observed between the survival (SV) and death (Death) groups in either cohort (training cohort: SV 24.4 ± 1.1 g vs. Death 24.5 ± 0.9 g, *P* = 0.155; validation cohort: SV 23.7 ± 0.8 g vs. Death 23.9 ± 0.3 g, *P* = 0.841) (Fig. 1D and E), effectively excluding the potential impact of body weight on irradiation outcomes. Furthermore, Pearson correlation analysis was performed to assess the relationship between initial body weight and post-irradiation survival time. The results revealed a non-significant weak correlation between body weight and post-irradiation survival time in both cohorts (training cohort: correlation coefficient *r*=-0.189, *p* = 0.149; validation cohort: correlation coefficient *r*=-0.305, *P* = 0.269), indicating that body weight had no effect on the survival time of mice after irradiation (Fig. 1F and G). Collectively, these findings demonstrate that the C57BL/6J mouse model established by 7.3 Gy ⁶⁰Co γ-ray whole-body irradiation can effectively evaluate individual differences in radiosensitivity.

### Cholesterol metabolism-related proteins as potential biomarkers for individual radiosensitivity in mice

**Fig. 2:**
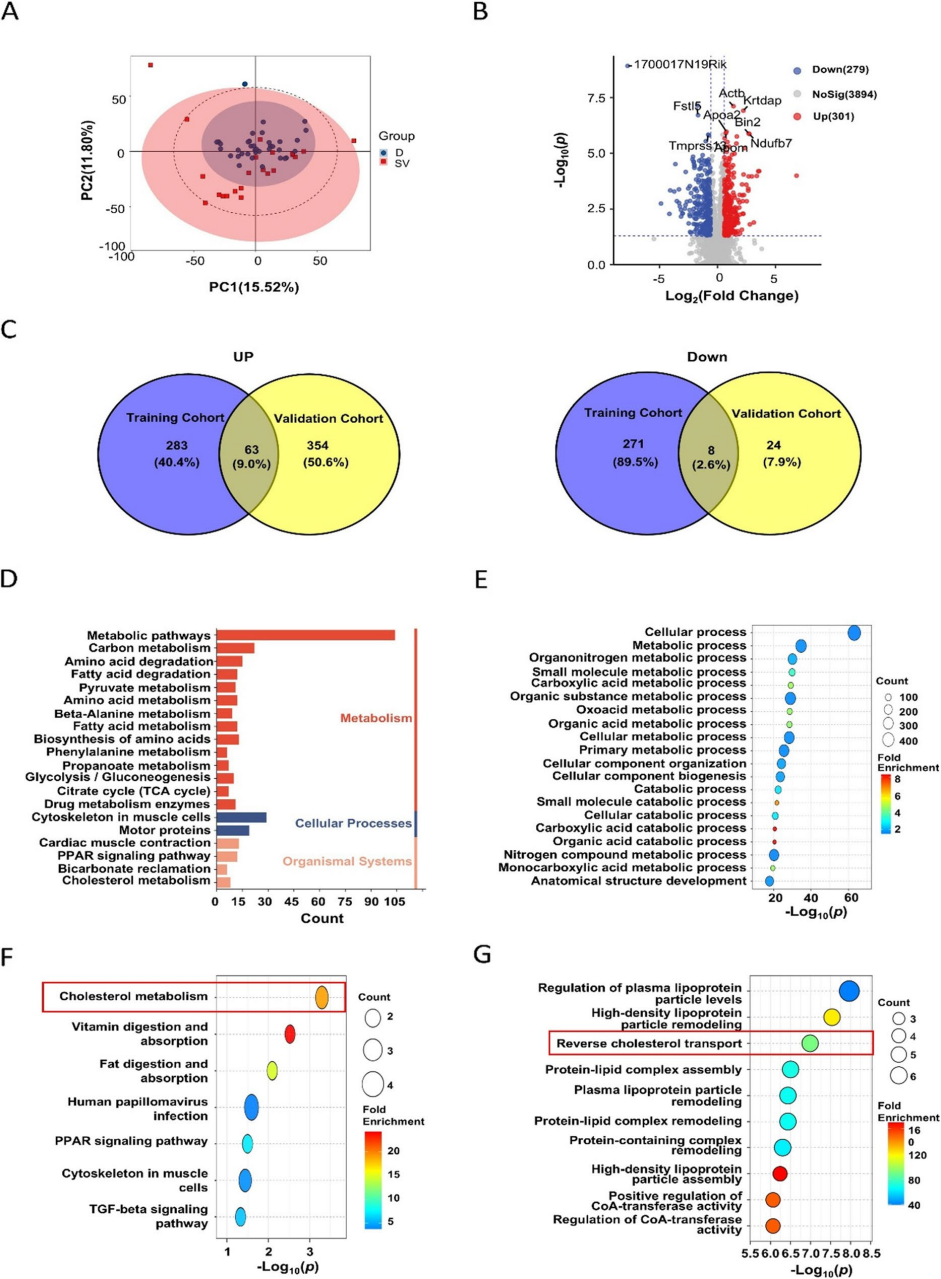
Differential protein analysis between lethal-sensitive and survival-resistant groups in the training and validation cohorts.‌ **A**.‌ Principal component analysis (PCA) of serum proteins from the survival-resistant group (red) and the lethal-sensitive group (blue) in the training cohort. Each point represents an individual sample. **B**.‌ Volcano plot of proteins differentially expressed between the radiation-resistant and radiation-sensitive groups in the training cohort. The vertical blue dashed lines indicate a ± 1.5-fold change threshold, and the horizontal dashed line indicates a p-value of 0.05. **C**.‌ Venn diagram showing the number of differentially expressed proteins (upregulated on the left, downregulated on the right) between the radiation-resistant and radiation-sensitive groups in the training (blue circle) and validation (yellow circle) cohorts. **D**.‌ Bar graph of the top 20 enriched KEGG pathways for the differential proteins between the lethal-sensitive and survival-resistant groups in the training cohort. The X-axis represents the number of differential proteins, and the Y-axis represents the KEGG pathway names. Colors correspond to pathway categories: red for Metabolism, light blue for Genetic Information Processing, green for Environmental Information Processing, dark blue for Cellular Processes, and pink for Organismal Systems. **E**.‌ Bubble plot of the top 20 enriched Gene Ontology (GO) biological process terms for the differential proteins in the training cohort. The X-axis is -log_10_(p-value) (a larger value indicates greater significance), and the Y-axis lists the GO terms. Bubble size represents the number of differential proteins (Count), and the color gradient represents the Fold Enrichment. **F**.‌ Bubble plot of KEGG pathway enrichment for the common differential proteins shared between the lethal-sensitive and survival-resistant groups in both the training and validation cohorts. The X-axis is -log_10_(p-value), and the Y-axis lists the KEGG pathways. Bubble size represents the number of differential proteins (Count), and the color gradient represents the Fold Enrichment. **G**.‌ Bubble plot of the top 10 enriched GO biological process terms for the common differential proteins shared between the training and validation cohorts. The X-axis is -log_10_(p-value), and the Y-axis lists the GO terms. Bubble size represents the number of differential proteins (Count), and the color gradient represents the Fold Enrichment

Based on data-independent acquisition (DIA)-based proteomic analysis of serum samples from the training cohort, a total of 5,799 proteins were identified. Principal Component Analysis (PCA) showed that the expression levels of differential proteins between mice in the radiation-sensitive (death) group and the radiation-resistant (survival) group were distinct and clustered separately (Fig. 2A), indicating that these differential proteins may be involved in individual radiosensitivity and have potential biomarker value. Further analysis of these differentially expressed proteins (DEPs) was performed using a volcano plot (Fig. 2B) and a heatmap (Supplementary Figure S1A). The results showed 580 statistically significant DEPs (*P* < 0.05, |log₂FC| ≥ 0.585) between the sensitive and resistant groups in the training cohort. Compared to the sensitive group, the resistant group exhibited 301 up-regulated and 279 down-regulated proteins (Supplementary Table S1). In the validation cohort, 449 DEPs were identified with statistical significance (*P* < 0.05, |log₂FC| ≥ 0.585) (Supplementary Figure S1B), with 417 up-regulated and 32 down-regulated proteins in the resistant group compared to the sensitive group (Supplementary Table S2). A comparative analysis between the two cohorts revealed 63 consistently up-regulated and 8 consistently down-regulated proteins in the resistant group compared to the sensitive group. These common DEPs may serve as potential biomarkers for individual radiosensitivity.

To identify differential proteins associated with individual radiosensitivity in mice, we performed Kyoto Encyclopedia of Genes and Genomes (KEGG) and Gene Ontology (GO) enrichment analyses on the differential proteins (*P* < 0.05, |log_2_FC| ≥ 0.585) from both the training and validation cohorts. KEGG pathway enrichment analysis indicated that metabolic pathways were significantly enriched in both cohorts (Fig. 2D, Supplementary Figure S1C). GO enrichment analysis showed that metabolism-related biological processes were highly significantly enriched, with a large number of involved differential proteins (Fig. 2E, Supplementary Figure S1D). This suggests that differential proteins involved in metabolic processes may be closely related to radiosensitivity. Further KEGG pathway enrichment analysis of the common differential proteins between the sensitive and resistant groups across both cohorts showed a primary enrichment in cholesterol metabolism pathways (Fig. 2F). Subsequent GO biological process enrichment analysis indicated that reverse cholesterol transport was significantly enriched, with a notable focus within biological processes related to plasma lipoprotein metabolism, including plasma lipoprotein particle regulation, remodeling, and other related processes (Fig. 2G). These findings suggest that proteins involved in cholesterol metabolism processes may be closely associated with individual radiosensitivity in mice and could serve as potential biomarkers for individual radiosensitivity.

### Robust performance of an ApoA1/ApoA2/ApoA4 biomarker panel in predicting individual radiosensitivity in mice

**Fig. 3:**
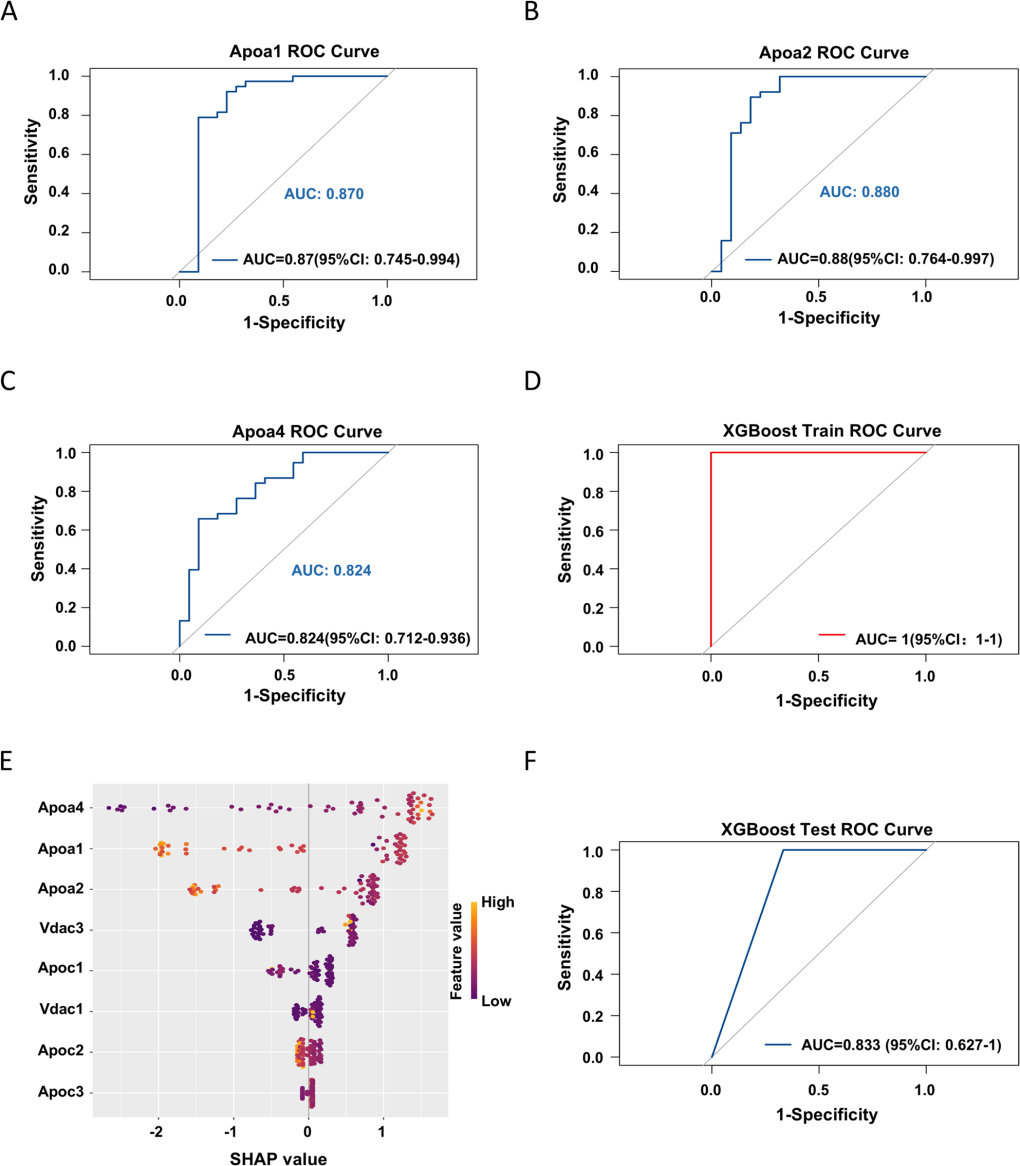
Predictive performance of an XGBoost model based on serum apolipoproteins for murine radiosensitivity. **A**-**C**.‌ Receiver operating characteristic (ROC) curves of ‌ApoA1‌, ‌ApoA2‌, and ‌ApoA4‌ for predicting radiosensitivity in the training cohort. ‌**D**.‌ ROC curve of the combined ‌ApoA1/A2/A4 panel‌ in the training cohort. ‌**E**. SHAP summary plot (beeswarm plot) for differential proteins enriched in the cholesterol metabolism pathway between radio-sensitive and radio-resistant groups in the training cohort. The y-axis represents protein names, and the x-axis represents the SHAP value (contribution): a positive value increases the prediction towards the death outcome (coded as 1), while a negative value decreases it (i.e., a larger negative value indicates a stronger contribution to survival). The color gradient from yellow to purple indicates protein expression levels (yellow: high, purple: low), allowing for intuitive observation of how concentration changes contribute to the outcome. The absolute value of the SHAP value signifies the magnitude of the protein’s impact on the model’s prediction. ‌**F**.‌ ROC curve of the combined ‌ApoA1/A2/A4 panel‌ in the validation cohort. For all ROC curves, the area under the curve (AUC) with its 95% confidence interval (CI) is annotated, serving as a metric for the model’s predictive performance. An AUC closer to 1 indicates a stronger discriminative ability

Given the association between cholesterol metabolism-related proteins and individual radiosensitivity, we developed an XGBoost machine learning model using cholesterol metabolism-related differential proteins identified in the training cohort. In the training cohort, the areas under the receiver operating characteristic curve (AUC) for apolipoproteins Apoa1, Apoa2, and Apoa4 in individually predicting mouse radiosensitivity were 0.870 (95% CI: 0.745–0.994), 0.880 (95% CI: 0.764–0.997), and 0.824 (95% CI: 0.712–0.936), respectively (Fig. 3A–C), indicating that each apolipoprotein possesses a certain predictive capability, with Apoa1 and Apoa2 demonstrating relatively superior performance. Following machine learning modeling of the combination of the three apolipoproteins using the XGBoost algorithm, the AUC value obtained in the training cohort reached 1 (95% CI: 1–1) (Fig. 3D), demonstrating a highly discriminative predictive ability of the combination. These results indicated that each of these individual serum protein markers exhibited high predictive efficacy, and their synergistic effects collectively accounted for the perfect AUC of the combined model.

To clarify the key roles of ApoA1, ApoA2, and ApoA4 in the prediction model, we calculated SHAP (SHapley Additive exPlanations) values for the cholesterol metabolism-related differential serum proteins in the training cohort to quantify their contribution to the prediction of individual sample radiosensitivity and interpret the XGBoost model outputs (Fig. 3E). The results revealed that the mean SHAP values of ApoA4, ApoA2, and ApoA1 were significantly higher than those of other proteins, indicating their more prominent predictive power. Specifically, ApoA1 and ApoA2 were identified as key features associated with radiation resistance; higher expression levels of these proteins corresponded to more negative SHAP values, suggesting a greater contribution to survival outcomes. In contrast, ApoA4 was a marker of radiation sensitivity, with higher expression levels associated with more positive SHAP values, indicating a greater contribution to mortality outcomes.

Furthermore, to further validate the predictive performance of the combination of these three apolipoproteins, in the independent validation cohort, the XGBoost model constructed based on this combination also performed excellently, with an area under the curve (AUC) of 0.833 (95% confidence interval [CI]: 0.627–1.000) (Fig. 3F), indicating that the model has favorable predictive discriminative power in independent validation.

Taken together, these results demonstrate that the robust predictive efficacy and good generalization ability of the ApoA1, ApoA2, and ApoA4 panel, underscoring its critical contribution to radiosensitivity prediction and significant potential as a novel clinical biomarker for this assessment.

### Robust association of serum ApoA1/ApoA2/ApoA4 levels with individual radiosensitivity in mice

**Fig. 4:**
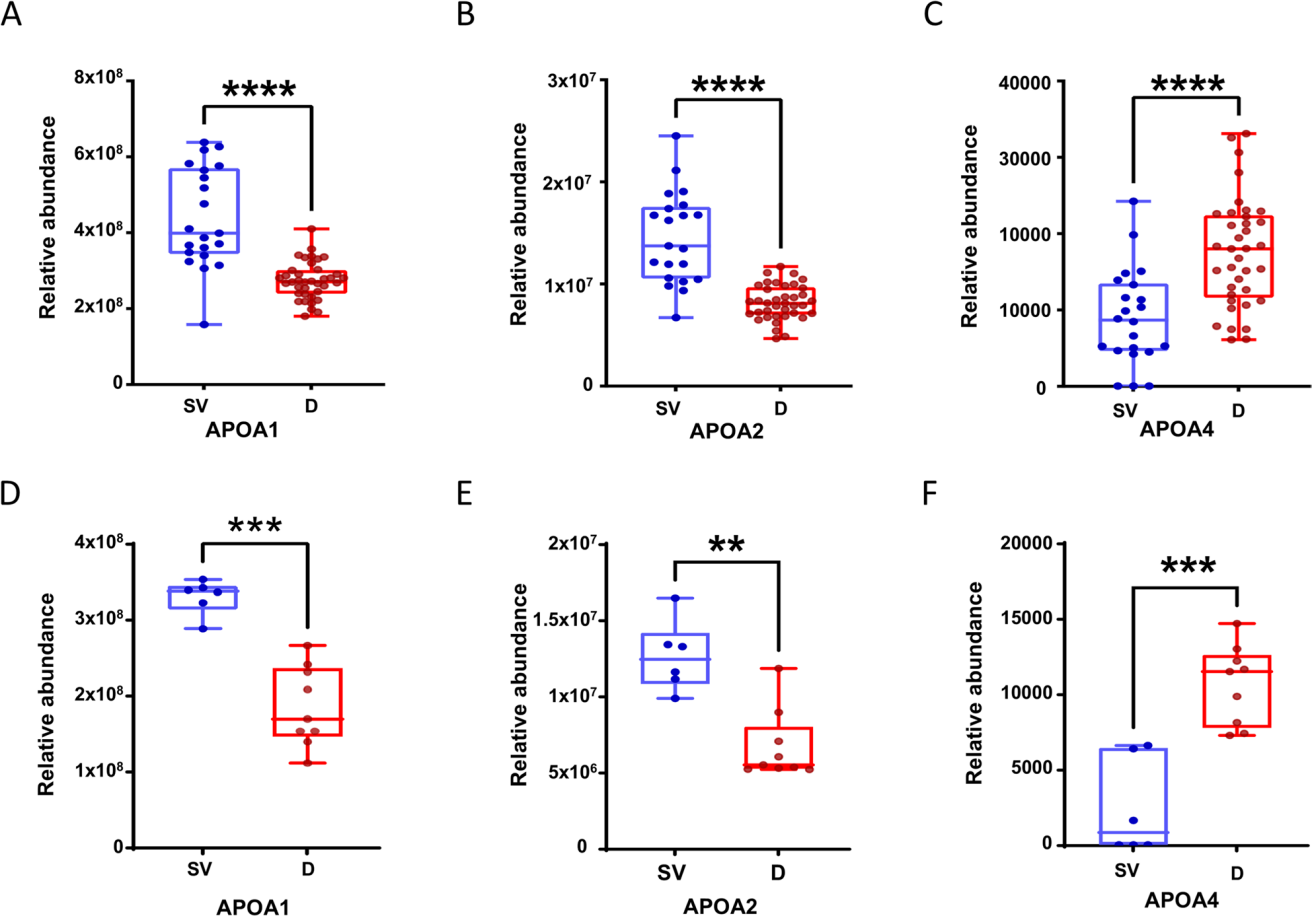
Expression levels of the serum apolipoprotein panel (ApoA1, ApoA2, ApoA4) in surviving and deceased mouse cohorts.‌ ‌**A**-**C**.‌ Box plots showing the relative expression of each apolipoprotein in the training cohort. ‌**D**-**F**. Box plots showing the relative expression of each apolipoprotein in the validation cohort. In each subplot, the x-axis indicates the experimental groups where “SV” represents the survival group and “D” represents the death group. The y-axis represents the relative expression level. Blue data points and box plots correspond to the SV group, while red data points and box plots correspond to the D group. The P-value (Student’s t-test) for the difference between the two groups is displayed above each subplot, with statistical significance denoted as *P < 0.05, ‌P < 0.01, *‌P < 0.001, ‌‌P < 0.0001. Box plots depict the median and interquartile range, and individual data points represent the measured values for each sample

To investigate the association between the expression levels of apolipoprotein family members (ApoA1, ApoA2, ApoA4) and individual radiosensitivity, we compared the mass spectrometry-based serum levels of these apolipoproteins between radiation-resistant (survivors) and radiation-sensitive (non-survivors) mice. In the training cohort, serum levels of ApoA1 and ApoA2 were significantly elevated in survivors compared to non-survivors (ApoA1: 4.2 ± 1.6 × 10⁸ vs. 2.7 ± 0.5 × 10⁸; ApoA2: 1.4 ± 0.5 × 10⁷ vs. 0.8 ± 0.2 × 10⁷; both *P* < 0.0001; Fig. 4A-B), suggesting a radioprotective role for these two apolipoproteins. Conversely, ApoA4 levels were markedly lower in survivors (9.0 ± 6.3 × 10³ vs. 18.0 ± 8.7 × 10³; *P* < 0.0001; Fig. 4C), implicating high ApoA4 expression in increased radiosensitivity. These findings were robustly validated in an independent validation cohort. The significant elevation of ApoA1 and ApoA2 in survivors was reconfirmed (both *P* < 0.01; Fig. 4D-E). For ApoA4, its expression trend remained entirely consistent with that observed in the training cohort, with levels significantly lower in survivors than in non-survivors (*P* < 0.001; Fig. 4F). Collectively, our analyses consistently demonstrate that high serum levels of ApoA1 and ApoA2 are associated with radiation resistance, whereas high serum ApoA4 levels are associated with radiation sensitivity—a finding that aligns with the results of our SHAP analysis.

### High ApoA1/ApoA2 and low ApoA4 expression as a serum protein signature for post-irradiation survival in mice

**Fig. 5:**
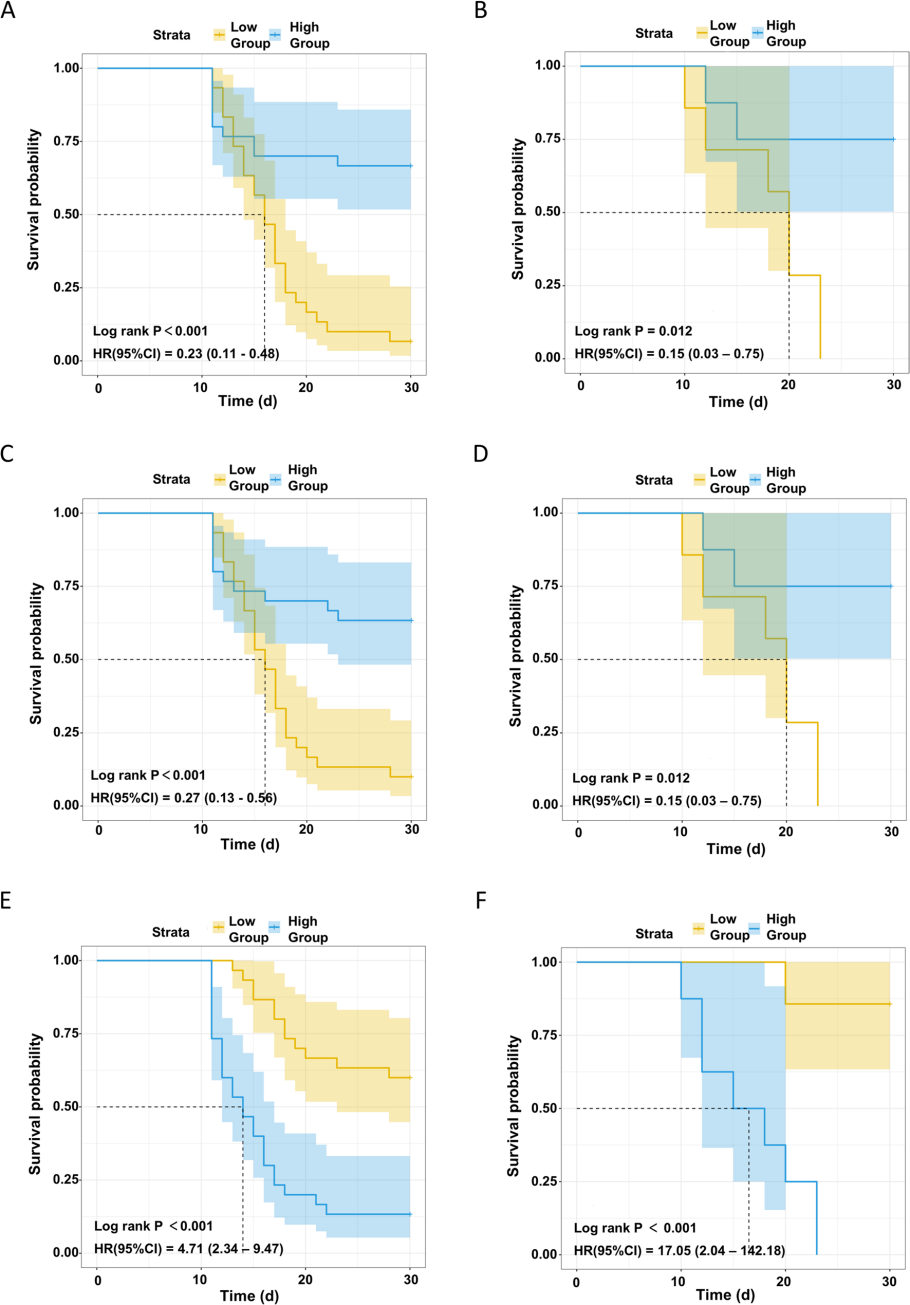
Cox proportional hazards regression model analysis of the impact of apolipoprotein ApoA1, ApoA2, and ApoA4 expression levels on survival outcomes after lethal irradiation.‌ ‌**A**-**B**. ApoA1‌: Kaplan-Meier survival curves for high vs. low ApoA1 expression in the training (**A**) and validation (**B**) cohorts. ‌**C**-**D**. ApoA2‌: Kaplan-Meier survival curves for high vs. low ApoA2 expression in the training (**C**) and validation (**D**) cohorts. ‌**E**-**F**. ApoA4‌: Kaplan-Meier survival curves for high vs. low ApoA4 expression in the training (**E**) and validation (**F**) cohorts. Each panel visually presents the probability of survival over time for groups with different expression levels. Yellow curves represent the low-expression group (Group = Low), and blue curves represent the high-expression group (Group = High). The Log-rank test P-value is used to determine if there is a statistically significant difference between the survival curves. The Hazard Ratio (HR) with 95% confidence interval (95% CI) quantifies the difference in survival risk between the high and low-expression groups. An HR < 1 indicates a lower risk of death (protective effect) for the high-expression group, whereas an HR > 1 indicates a higher risk (detrimental effect), thereby assessing the impact of each apolipoprotein on survival outcomes

To further investigate the association of ApoA1, ApoA2, and ApoA4 with survival outcomes after lethal irradiation, we stratified mice by median pre-irradiation serum levels into high- and low-expression groups for Kaplan-Meier analysis. The hazard ratio (HR) was assessed using the Cox proportional hazards model (Fig. 5). In both the training and validation cohorts, high expression of ApoA1 or ApoA2 was significantly associated with improved survival (Log-rank *P* < 0.05 for all; Fig. 5A-D). The HR with a 95% confidence interval (CI) was less than 1, confirming ApoA1 and ApoA2 as independent protective factors associated with a significantly reduced risk of death. In stark contrast, high ApoA4 expression predicted significantly poorer survival (training cohort: HR = 4.71, 95% CI: 2.34–9.47; log-rank *P* < 0.001). This adverse association was even more pronounced in the validation cohort, with a strikingly higher HR of 17.05 (95% CI: 2.04–142.18; log-rank *P* < 0.001) (Fig. 5E, F). In summary, these survival analyses corroborate our previous findings, establishing high ApoA1/ApoA2 and low ApoA4 as a robust serum protein signature predictive of survival following lethal irradiation.

### Serum ApoA4 serves as a potential biomarker for predicting extreme radiation risk in mice

**Fig. 6:**
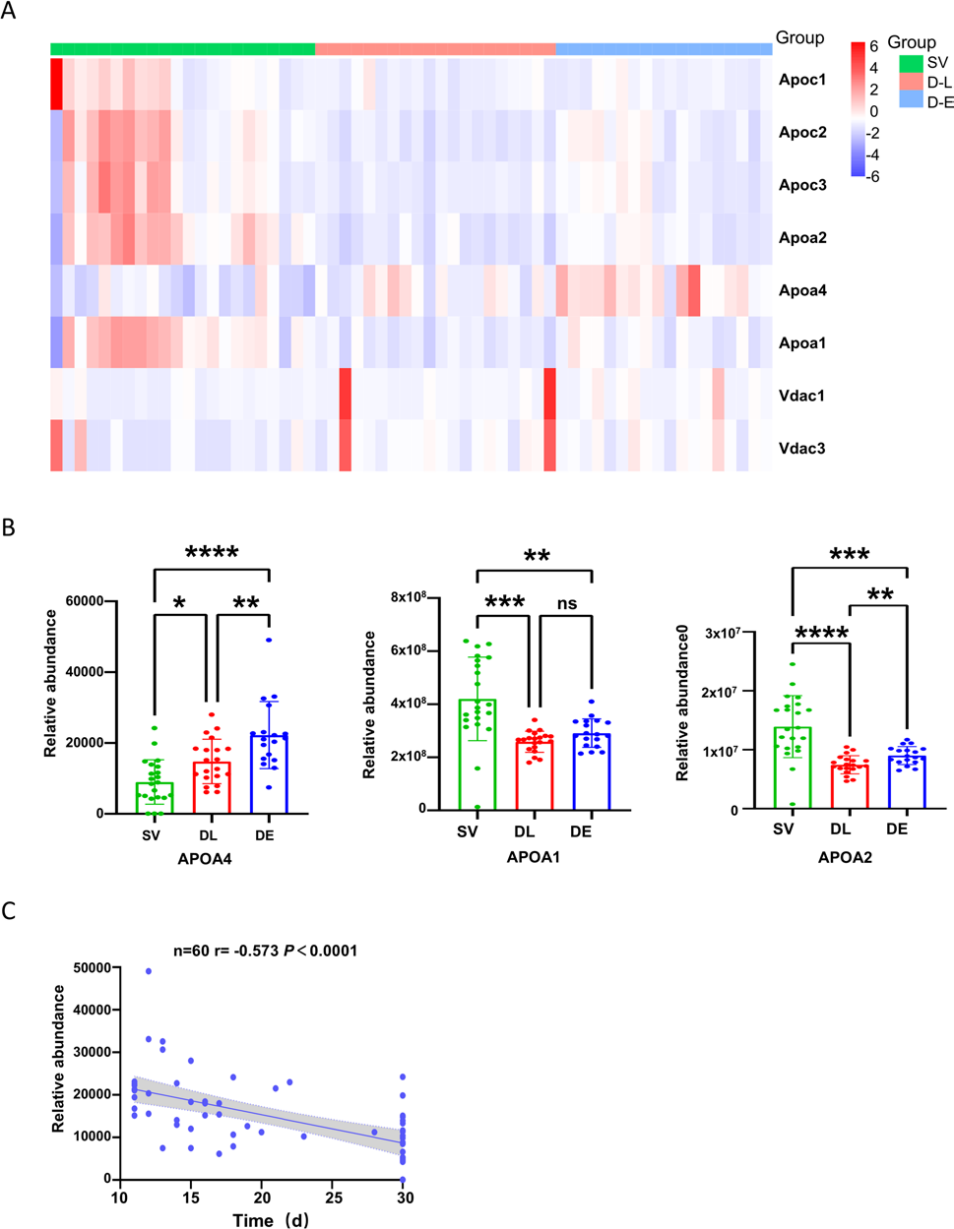
Association between serum ApoA4 expression and post-radiation outcomes.‌‌ ‌**A**.‌ Heatmap of differential cholesterol metabolism-related serum protein expression in the training cohort. Rows represent different proteins (e.g., ApoA1, ApoA2, ApoA4), and columns represent samples from the early-death (D-E, survival days < 15), late-death (D-L, 15 ≤ survival days ≤ 30), and survival (SV, survival days ≥ 30) groups. Color intensity indicates relative expression levels (blue: low expression, red: high expression). ‌**B**.‌ Bar plot showing ApoA1/A2/A4 expression levels in the early-death (*n* = 18), late-death (*n* = 20), and survival (*n* = 22) groups. Data are presented as mean ± SD. Multiple comparison tests were used for multiple group comparisons. P value < 0.05 was considered statistically significant. Significance levels: ‌****P < 0.0001, ***‌P < 0.001, **P < 0.01, *P < 0.05. ‌**C**.‌ Pearson correlation scatter plot between ApoA4 expression levels and post-radiation survival time. *n* = 60, r denotes the correlation coefficient. *P* < 0.0001 indicates the difference is statistically significant

The aforementioned results suggest that serum proteins within the cholesterol metabolism pathway may be closely associated with individual radiosensitivity. To identify potential biomarkers for predicting extreme radiation risk, we performed a detailed analysis of these proteins in the training cohort, specifically examining their association with early death (≤ 15 days), late death (> 15 days), and survival outcomes (Fig. 6). Among the proteins analyzed, apolipoprotein A4 was the only protein that demonstrated a significant association between its expression levels and altered time to death in mice (*P* < 0.01). In contrast, other proteins, including apolipoprotein A1 and apolipoprotein A2, did not show consistent associations—although reduced expression of these proteins was linked to delayed death, further reduction did not lead to earlier mortality (Fig. 6A, B). ‌To further characterize this relationship, we performed Pearson correlation analysis between pre-irradiation serum ApoA4 levels and actual survival time, which established a significant negative correlation (*n* = 60, *r*=-0.573, *P* < 0.0001; Fig. 6C). This finding directly links elevated ApoA4 expression to increased radiosensitivity and shorter survival. This confirms ApoA4’s specific role in predicting extreme risk.

## Discussion

Given the significant inter-individual variability in radiation sensitivity, accurate prediction plays a vital role in protecting the health of radiation professionals, refining cancer radiotherapy strategies, and evaluating the radiation hazards encountered by astronauts in space exploration. Despite advancements in genomic testing technologies that enable the evaluation of individual radiosensitivity, their high cost and poor timeliness have severely limited their clinical application [5]. Therefore, the development of convenient and effective predictive biomarkers has become a current research priority. Serum proteins have emerged as ideal candidate biomarkers due to their advantages of non-invasive, convenient detection and dynamic monitoring [19]. Notably, serum apolipoprotein levels, by dynamically regulating cholesterol transport, can influence DNA damage repair efficiency and redox balance, ultimately affecting radiosensitivity [20]. Nevertheless, whether apolipoproteins can serve as effective biomarkers for predicting individual radiosensitivity remains unclear. This study establishes, for the first time, that a serum apolipoprotein panel (ApoA1/ApoA2/ApoA4) serves as an effective biomarker for predicting individual radiosensitivity. The predictive model exhibited robust predictive performance in a training cohort and strong generalizability in an independent validation cohort, thereby providing a novel molecular tool and technical support for the precise stratification of radiation risk in nuclear occupational groups and the optimization of personalized radiotherapy regimens for tumors.

Cholesterol metabolic homeostasis, maintained through a regulated network of biosynthesis, uptake, efflux, and transport, is integral to cellular function and organismal homeostasis [21]. Accumulating evidence indicates that cholesterol and its metabolites contribute critically to tumor radioresistance [22–24]. For instance, Jin et al. observed that exogenous cholesterol supplementation enhances radioresistance in colorectal cancer cells, whereas blocking the SREBP-1/FASN pathway to inhibit cholesterol synthesis significantly increased radiosensitivity by suppressing proliferation and inducing apoptosis [25]. These findings collectively reveal a close association between cholesterol metabolism and radiosensitivity, but the underlying mechanisms of its role in individual radiosensitivity differences remain to be systematically elucidated. Through comparative proteomic analysis of survival versus death groups in a 7.3 Gy half-lethal dose mouse model, we identified the cholesterol metabolism pathway as a key determinant of post-irradiation fate. This conclusion is supported by a recent study by Liu et al., who demonstrated that cholesterol enhances ferroptosis resistance in hematopoietic stem cells by activating the SLC38A9-mTOR signaling axis, thereby promoting myeloid differentiation and self-renewal, ultimately improving survival in mice with lethal radiation-induced myelosuppression [26]. Notably, our GO enrichment analysis revealed that the biological process of cholesterol transport—rather than its biosynthesis—may play a central role in determining radiation outcomes. This discovery expands our current understanding of the role of cholesterol metabolism in individual radiosensitivity, suggesting that the spatial distribution and transport efficiency of cholesterol may also have important biological significance. Future studies should employ gene-editing tools to validate the key roles of cholesterol transport-related genes and ultimately develop novel radioprotective strategies targeting this process.

Apolipoproteins are key components of serum lipoproteins, critical for cholesterol transport and metabolism, and play important roles in biological processes and pathophysiological pathways including diabetes, cancer, and obesity [27]. As the core component of high-density lipoprotein (HDL), apolipoprotein A family (notably ApoA1, ApoA2, ApoA4 and ApoA5) removes excess arterial cholesterol via reverse cholesterol transport for hepatic metabolism or excretion [28]. Among ApoA family members, ApoA1 dominates HDL’s protective functions, mediating cardiovascular protection through promoting reverse cholesterol transport and anti-inflammatory effects, while ApoA2 and ApoA4 exert synergistic roles in cholesterol metabolism, vascular integrity, and immune regulation [29–31]. Notably, their unique roles in health and disease highlight their potential as biomarkers and therapeutic targets. For instance, ApoA1 serves as an early diagnostic biomarker for Alzheimer’s disease [32]; ApoA2 is a serum biomarker for metastatic renal cell carcinoma [33]; and elevated ApoA4 in a mouse model of middle cerebral artery occlusion indicates its potential for stroke [34]. Furthermore, a limited number of studies also suggest ApoA proteins as prognostic biomarkers for tumor radiotherapy: higher pre-treatment ApoA1 correlates with better overall survival in small cell lung cancer patients receiving thoracic radiotherapy [35]. However, their value as biomarkers for individual radiosensitivity remains unreported. This study first demonstrated that high ApoA1/ApoA2 expression confers a survival advantage in irradiated mice, whereas high ApoA4 is a mortality risk factor. This three-protein panel exhibited excellent performance in predicting post-irradiation survival in both training and independent validation cohorts. From a perspective of functional coherence, the elevated expression of ApoA1 and ApoA2 in the survival group suggests their potential involvement in regulating radioresistance, whereas the low expression of ApoA4 in the survival group indicates its potential role in promoting radiosensitivity. Although direct experimental evidence for their role in modulating radiosensitivity is currently lacking, existing studies indicate that the protective roles of ApoA1/A2 may be linked to their anti-inflammatory and antioxidant properties, whereas the risk associated with ApoA4 may stem from its context-dependent pro-inflammatory effects [36–39]. Our study establishes the first strong association between this ApoA panel and radiation outcomes, providing a theoretical basis for translational application in radiation injury risk assessment and radiosensitivity prediction. Subsequent studies should clarify the direct regulatory effects of ApoA1/2/4 on radiosensitivity in vitro/in vivo and decipher their downstream molecular mechanisms.

In addition, the present study found that serum ApoA4 levels in the early death group were significantly higher than those in the late death/survival group, indicating its potential to identify individuals at extremely high risk of mortality. As a multifunctional regulator of inflammation, lipid metabolism and hepatic homeostasis, the marked elevation of pre-radiation ApoA4 may indicate pre-existing pathological vulnerability in the host, which is further exacerbated upon radiation exposure. Specifically, this elevation may reflect a pre-activated systemic inflammatory state—pro-inflammatory cytokines (e.g., IL-6, TNF-α), which are closely associated with radiation-induced immunopathology, can dysregulate intestinal ApoA4 secretion [40], thereby depleting the host’s anti-inflammatory reserves and impairing its ability to counteract radiation-triggered reactive oxygen species generation and NF-κB activation [41]. Moreover, abnormal serum ApoA4 levels often indicate subclinical hepatic dysfunction [42, 43], which exacerbates radiation-induced dyslipidemia (e.g., elevated triglycerides, reduced cholesterol esters) [44] and associated oxidative stress [45]. Meanwhile, increased ApoA4 levels may also reflect pre-existing defects in host lipid transport [46]. Such defects are further aggravated by radiation-induced inhibition of fatty acid oxidation and promotion of lipolysis [47], leading to lipid overload, which in turn enhances ROS production and tissue damage [48]. In conclusion, elevated ApoA4 levels are likely not merely a passive disease marker, but rather an integrated indicator reflecting interconnected pathological vulnerabilities including pre-activated inflammation, subclinical hepatic dysfunction and lipid dysregulation. These vulnerabilities are amplified under radiation exposure, ultimately contributing to early mortality.

## Conclusions

This study is the first to identify a panel of serum apolipoproteins (ApoA1, ApoA2, ApoA4) as novel minimally invasive biomarkers for predicting individual radiosensitivity in mice. The constructed XGBoost predictive model exhibited excellent performance and robust generalizability, while Cox proportional hazards survival analysis further revealed distinct associations between specific apolipoproteins and post-irradiation survival outcomes—with ApoA1/ApoA2 serving as protective factors and ApoA4 as an adverse prognostic indicator. This panel suggests a potential link between cholesterol metabolism and radiation response, thereby offering novel insights to advance personalized radiation protection and therapeutic strategies. Given the limitations of the animal model, future research should verify the panel’s clinical predictive efficacy, elucidate its underlying molecular mechanisms, and explore the translational potential of this apolipoprotein panel for personalized radiation protection and precise radiotherapy.

## Supplementary Information

Supplementary Material 1.

Supplementary Material 2.

## Data Availability

The original contributions presented in this study are included in the article. Further inquiries can be directed to the corresponding author.

## Abbreviations

APOA: Apolipoprotein A
APOA1: Apolipoprotein A1
APOA2: Apolipoprotein A2
APOA4: Apolipoprotein A4
APOA5: Apolipoprotein A5
APOE: Apolipoprotein E
AUC: Area Under the Curve
BP: Biological Process
CI: Confidence Interval
DIA: Data-Independent Acquisition
DEP: Differentially Expressed Protein
FDR: False Discovery Rate
FC: Fold Change
HDL: High-Density Lipoprotein
HR: Hazard Ratio
PCA: Principal Component Analysis
ROC: Receiver Operating Characteristic
SHAP: SHapley Additive exPlanations
XGBoost: eXtreme Gradient Boosting
XRCC1: X-ray Repair Cross-Complementing Group 1
XRCC3: X-ray Repair Cross-Complementing Group 3
