# Supplementary material for: Predictive value of serum apolipoprotein panel (ApoA1 / ApoA2 / ApoA4) as a biomarker for individual radiosensitivity
Source: Lipids Health Dis. 2026 Jan 26;25:61. doi: 10.1186/s12944-026-02868-8 (PMC12918045; doi:10.1186/s12944-026-02868-8)
Supplement: Supplementary file 2 — Supplementary Material 2. [file 12944_2026_2868_MOESM2_ESM.docx]

**Supplementary Materials**

**Legends to supplementary Figures**

**Supplementary Figure S1**


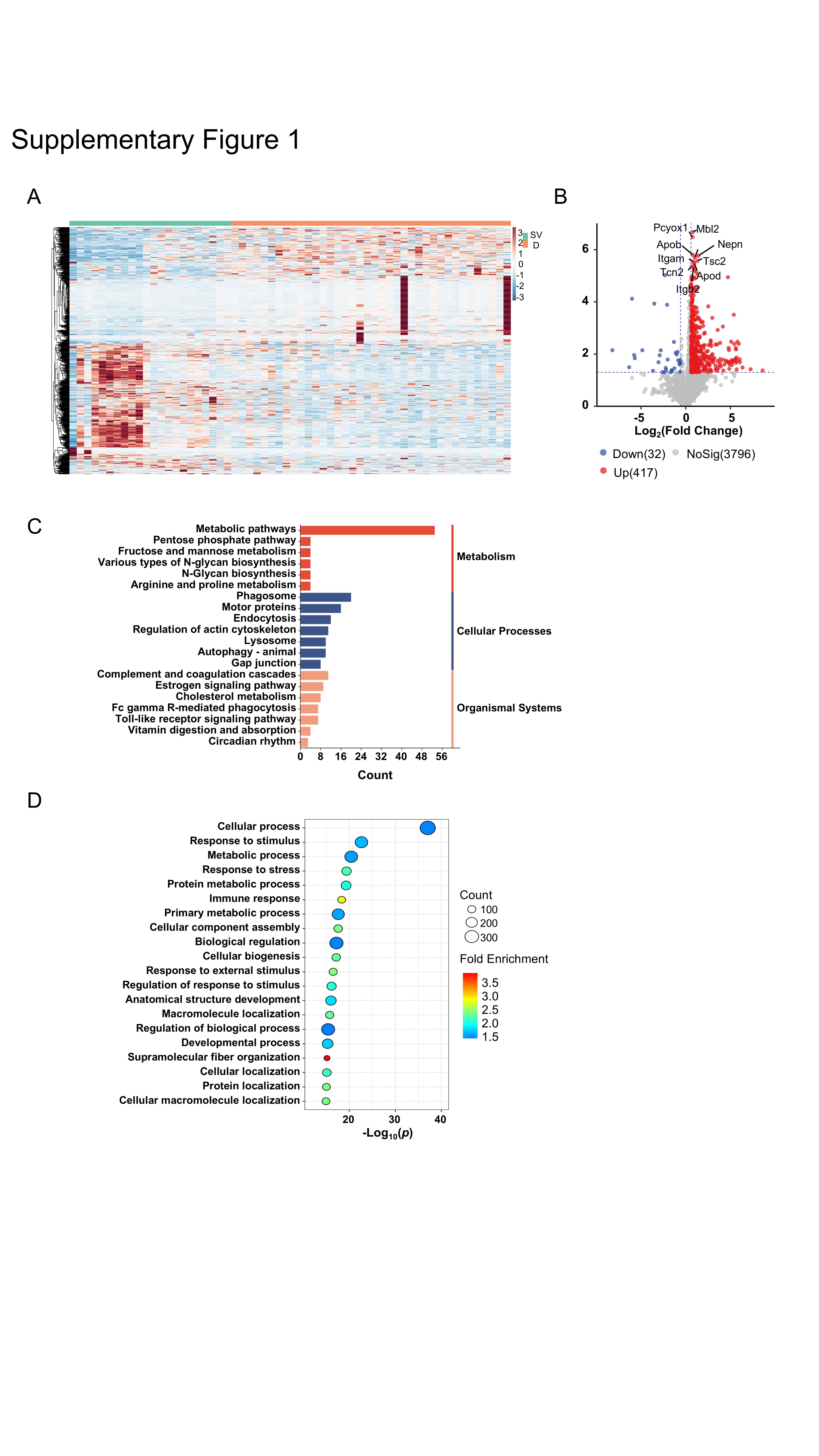


**Supplementary Figure S1. Differential protein analysis between radiation sensitive (death) and resistant (survival) mouse groups in training and validation cohorts.** A Heatmap showing the expression levels of differentially expressed proteins (DEPs) identified from DIA-based proteomic analysis of serum protein samples in the training cohort. The color scale represents relative protein expression levels, and samples are grouped by radiation lethality sensitivity (D2) and resistance (SV2). B Volcano plot of proteins differentially expressed between the radiation-resistant and radiation-sensitive groups in the validation cohort. The vertical blue dashed lines indicate a ±1.5-fold change threshold, and the horizontal dashed line indicates a p-value of 0.05. C Bar chart of top 20 KEGG pathway enrichments for DEPs in the validation cohort. The X-axis represents the number of DEPs, and the Y-axis represents KEGG pathway names. Different colors correspond to pathway categories: red for Metabolism, light blue for Genetic Information Processing, green for Environmental Information Processing, dark blue for Cellular Processes, and pink for Organismal Systems. D Bubble plot of top 20 GO biological process enrichments for DEPs in the validation cohort. The X-axis is -Log₁₀(p) (higher values indicate more significant enrichment), and the Y-axis represents GO biological process terms. Bubble size indicates the number of DEPs (Count), and color gradient represents Fold Enrichment (darker colors indicate higher fold enrichment).

**Supplementary Figure S2**

**Supplementary Figure S2. Kaplan-Meier survival curves of C57BL/6J mice after whole-body ⁶⁰Co γ-ray irradiation.** The curves correspond to three distinct irradiation dose groups (10 mice per group): the 7 Gy group in blue, the 7.5 Gy group in red, and the 8 Gy group in green. By the 30-day observation endpoint post-irradiation, the survival rates of the 7 Gy, 7.5 Gy, and 8 Gy groups were 70%, 20%, and 0% respectively.
